# Fucoidan as a Promising Drug for Pain Treatment: Systematic Review and Meta-Analysis

**DOI:** 10.3390/md22070290

**Published:** 2024-06-24

**Authors:** Miguel Á. Huerta, Miguel Á. Tejada, Francisco R. Nieto

**Affiliations:** 1 Department of Pharmacology, University of Granada, 18016 Granada, Spain; huerta@ugr.es (M.Á.H.); mtejada@ugr.es (M.Á.T.); 2 Institute of Neuroscience, Biomedical Research Center, University of Granada, 18016 Granada, Spain; 3 Biosanitary Research Institute ibs.GRANADA, 18012 Granada, Spain

**Keywords:** polysaccharides, leukocyte, analgesia, drug discovery, inflammation, immunity

## Abstract

Fucoidan is a polymer of L-fucose and L-fucose-4-sulphate naturally found in marine sources that inhibits p-selectin, preventing neutrophil recruitment to the site of injury. Fucoidan is employed in many studies as a tool to investigate the contribution of neutrophils to pain, showing analgesic effects. We performed a systematic review and meta-analysis to quantify the analgesic effects of pretreatment with fucoidan reported in the available preclinical studies. In addition, we summarized the articles which have studied the therapeutic effects of fucoidan in pathological pain at preclinical and clinical levels. The results of this systematic review reveal that pretreatment with fucoidan is a powerful tool which reduces neutrophil infiltration by 70–90% at early time points. This meta-analysis showed that preventative treatment with fucoidan produced a significant pain reduction. In addition, several preclinical studies have observed that fucoidan treatment reduces the pain that is associated with various pathologies. Finally, fucoidan has also been tested in several clinical trials, with some degree of analgesic efficacy, but they were mostly small pilot studies. Considering all the above information, it can be concluded that fucoidan is not only a preclinical tool for studying the role of neutrophils in pain but also a promising therapeutic strategy for pain treatment.

## 1. Introduction

The neutrophil is the first immune cell to respond to tissue damage or a potential aggressor [1]; in response to an injury, it migrates massively to the inflammatory focus, being clearly the most abundant cell [2]. Its role there is fundamental: on the one hand, thanks to its phagocytic capacity, it allows for the clearance of cellular debris associated with tissue damage and pathogens, along with degranulation and the release of nuclear material in the form of neutrophil extracellular traps (NETs), which also promote this process; on the other hand, it has the ability to release pro-inflammatory signaling molecules (mainly cytokines) that “call” other cells, favoring and accelerating their activation and migration to the affected area [3,4,5]. It can also sensitize sensory neurons, promoting their discharge [6,7]. These varied actions of neutrophils could participate in pain modulation.

The role of neutrophils in pain has been extensively studied in experimental research. In several studies, including early ones on the topic [8,9,10], a pronociceptive role has been proposed, contributing to the generation of hyperalgesia and allodynia in response to various stimuli [11,12,13]. Other authors demonstrate that under certain circumstances, neutrophils can produce analgesia [14,15,16,17], mainly associated with the production and release of endogenous opioid peptides (EOPs), such as β-endorphin, enkephalins, dynorphins, and endomorphins [14,18]. Additionally, some studies take a middle ground and argue that neutrophils do not contribute to pain [19,20]. Understanding the role of this immune population in pain could be of significant interest to the medical and scientific community, as it could lead to new therapeutic targets for pain of different etiologies and improve the pathophysiological understanding of pain and its neuroimmune modulation.

A significant portion of studies investigating the contribution of neutrophils to pain in animal models employ a strategy based on neutrophil depletion using pharmacological treatment. Immunological strategies are the standard treatment for achieving neutrophil depletion. In early studies, at the end of the century, anti-neutrophil sera were used [17,21,22]. A few years later, with the advent of monoclonal antibodies, the anti-Gr-1 antibody (RB6-8C5) was used, which achieves robust depletion but is not selective for neutrophils (it affects other immune cells, mainly monocytes) [20,23,24]. In contrast, the anti-Ly6G antibody has superior selectivity and generally only affects neutrophils [25,26,27], although some studies report a significant reduction in macrophages [11]. However, these strategies with biological treatments have several limitations [28]: (1) as they are generally new molecules with high production costs, the cost is high; (2) it has been reported that the potent and sudden depletion produced by these treatments can generate a rebound response where there is a prominent proliferation in the bone marrow to try to compensate for this deficiency state [29,30]; (3) because the same markers (GR-1 and Ly6-G) are used for the detection and quantification of neutrophils, there may be antigen masking, leading to misinterpretation of a good depletion efficiency [30]; (4) incomplete depletion of neutrophils [29]; and (5) organ- and strain-specific neutrophil depletion [28]. Based on these drawbacks, some authors have opted to use fucoidan to evaluate the role of neutrophils in their pain models.

In addition to being a tool for studying neuroimmune interactions in pain, some authors propose the use of fucoidan as a therapeutic strategy for pain related with several conditions [31,32]. Furthermore, fucoidan has also been proposed for blocking post-ischemic reperfusion injury, in which the cascade of inflammation causes tissue damage [33,34,35]. Although these activities are mainly related with the inhibition of neutrophil recruitment, other mechanisms of fucoidan may contribute to the analgesic and anti-inflammatory properties: selective inhibition of COX-2 [36], inhibition of hyaluronidase [36,37], mitogen-activated protein kinase p38 inhibition [38], protein denaturation inhibition [39], and stabilization of the cell membrane integrity of human red blood corpuscles [39]. Several articles evaluated the analgesic efficacy of fucoidan in different models of pain, and even a few clinical experiences have been reported [40]. Therefore, the number of studies evaluating fucoidan for various purposes has significantly increased in recent years [41].

The objective of this work is to gather all the studies that employ fucoidan to investigate the role of neutrophils in animal models of pain and quantify the effect over pain. For this purpose, we will perform a systematic review and meta-analysis to quantify the analgesic effects of pretreatment with fucoidan reported in the available preclinical studies. Additionally, we will also gather studies proposing fucoidan as a therapeutic strategy for pain associated with different pathological conditions, both at preclinical and clinical level.

## 2. Results and Discussion

### 2.1. Fucoidan: Mechanism of Action

Fucoidan (also called fucoidin) is a polymer of L-fucose and L-fucose-4-sulphate which belongs to a group of sugar analogs [42]. It is found naturally in marine sources, mainly in brown seaweeds (e.g., *Fucus vesiculosus*, *Cladosiphon* sp., or *Undaria* sp.), and some marine invertebrate tissues [43,44]. It is relevant to note that fucoidan abundance and composition may vary between these sources and even between growing conditions, geographic location, harvesting season, anatomical regions, and extraction procedures [41,42,45,46,47,48,49,50,51]. The mechanism of action of fucoidan on immune cells is based on the inhibition of cell adhesion molecules, P-selectin, and to a lesser extent, L-selectin [52,53,54,55,56]. Both are highly abundant on the neutrophil membrane and are necessary for their adhesion/extravasation during an acute inflammatory response [57]. P-selectin also promotes neutrophil extracellular trap formation [58]. However, these proteins are not exclusive to neutrophils and are found in other immune cell populations (e.g., macrophages [59], or lymphocytes [60]). Therefore, fucoidan treatment may directly affect the migration of other immune cells to the site of inflammation, such as macrophages/monocytes or lymphocytes [61]. Nevertheless, most models assessing the role of fucoidan on pain were acute models, where the pain response was generally evaluated in a short period after pain induction (e.g., 0.5–3 h after inflammatory substance injection [62,63,64,65,66,67,68]), when neutrophil migration is very prominent and the presence of other populations is residual. Therefore, fucoidan analgesic effects observed by these studies probably mainly account for neutrophil migration inhibition. In this sense, fucoidan can be considered appropriate for evaluating the contribution of neutrophils in these acute inflammatory models.

### 2.2. Fucoidan: A Tool to Study the Role of Neutrophils in Pain

**Figure 1:**
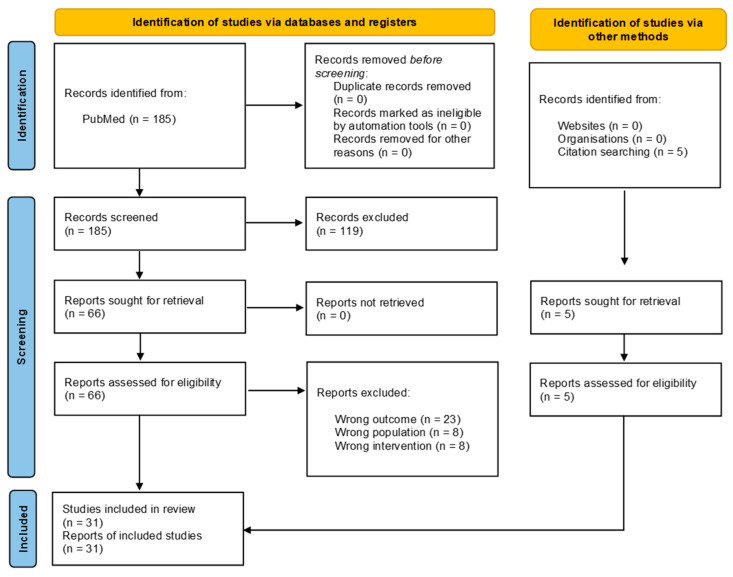
Study selection PRISMA flow diagram.

The search results and selection strategy are detailed in the PRISMA flowchart (Figure 1). In total, 31 articles met the inclusion criteria, evaluating the role of neutrophils in pain using pretreatment with fucoidan as a tool. The main information of these articles is summarized in Table 1. All the evaluations were performed on rats (16/31/) or mice (15/31). The doses administered ranged between 10 and 100 mg/kg, and the administration time before injury was 10–30 min in most of the studies. Pain models evaluated were diverse. In majority, inflammation was induced by an intraplantar injection of complex pro-inflammatory substances (CFA [69,70] or carrageenan [63,65,68]), or isolated pro-inflammatory compounds such as 5-HT [71,72], TNF [64,73], IL-17 [73], and others [62,63,74,75,76]. The other studies employed arthritis models with an intra-articular administration of inflammatory substances such as zymosan [77], mBSA [78,79], LTB4 [77], IL-17 [79], MSU crystals [80,81], and others [82,83,84,85,86]. Moreover, two articles evaluated muscle pain [66,87], and one employed a neuropathic pain model (postherpetic neuralgia [88]). On the other hand, pain was mainly assessed by the evaluation of reflex-based responses to stimuli (evoked pain)—mechanical hyperalgesia, mechanical allodynia, and heat hyperalgesia—although non-evoked nociceptive behavior was also explored in some articles. The assessment of non-evoked pain has been suggested to be more translational as it better reflects the clinical situation of patients in which spontaneous pain is more clinically relevant than cutaneous hypersensitivity [89].

### 2.3. Efficacy of Fucoidan as a Neutrophil Depletion Strategy

The efficacy of neutrophil depletion was assessed by diverse quantification methods: immunohistochemistry, flow cytometry, or myeloperoxidase were the most common (see Table 1). Most of them (≈90%) employed the myeloperoxidase assay (MPO), which allows estimating the number of neutrophils per mg of tissue. Although this technique demonstrated a high correlation with the number of neutrophils [85], the enzyme is also present in monocytes/macrophages [95], so the quantification might not be totally selective for neutrophils. In contrast, quantification using flow cytometry is much more selective as specific markers are used to differentiate populations, yet the methodology is more complex [94]. However, the oldest studies employed dyes followed by a manual counting [69], which can be considered a semi-quantitative method. As an example, the oldest article included in this analysis observed that pretreatment with fucoidan induced a slight decrease (40%) in the number of immune cells containing β-endorphin (mainly neutrophils) after CFA injection [69]. This low efficacy observed can be associated with a too low of a dose being used (10 mg/kg) [69]. On the contrary, all the studies published thereafter employed higher doses (20–40 mg/kg), achieving a great neutrophil depletion efficacy (70–90%).

The use of fucoidan for neutrophil depletion presents several advantages: (1) it only blocks cell extravasation and does not affect the blood count of neutrophils [61], while other strategies such as antibodies anti-Ly6G (1A8) or anti-Gr-1 (RB6) also reduce blood-circulating neutrophils [28,96]; (2) fucoidan does not affect neutrophil tissular levels in naive animals, while antibodies cause a robust reduction observed in different organs (spleen, lungs or bone marrow) [28,97]. Moreover, neutrophil depletion has been associated with a decrease of edema in the injured area in several models [98,99]. Based on all the above, it can be concluded that preventive treatment with fucoidan is effective for neutrophil depletion and presents some advantages in comparison to other methods.

### 2.4. Analgesic Efficacy of Preventive Treatment with Fucoidan

In all the articles compiled in Table 1, the effect of pretreatment with fucoidan on pain is evaluated. As mentioned in the previous section, in all cases, neutrophil depletion was robust (generally, a reduction of 70–90%). In 95% of the studies, a significant and robust reduction in pain was observed. It is likely that the absence of an analgesic effect of fucoidan observed in some of the works was due to the dose of fucoidan being too low (e.g., 10 mg/kg [69]).

**Figure 2:**
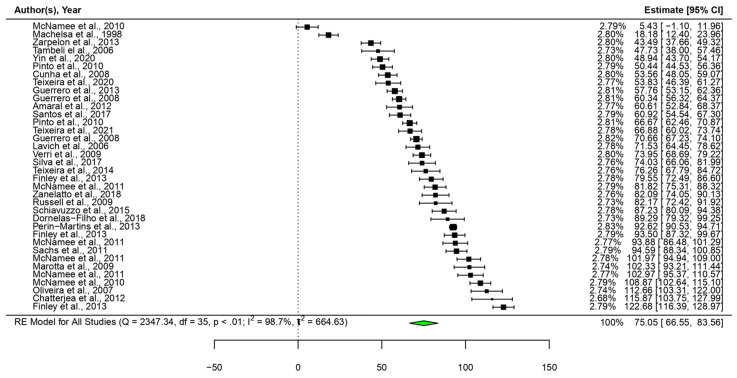
Forest plot of the pain reduction efficacy of pretreatment with fucoidan. CI, confidence interval; RE, random effects [62,63,64,65,66,68,69,71,72,73,74,75,76,77,78,79,80,82,85,86,87,90,91,92,94].

Next, the analgesic efficacy of neutrophil depletion with fucoidan was quantified by conducting a meta-analysis (Figure 2). Fucoidan clearly showed significant analgesic efficacy (*p* < 0.0001) with a large effect size (75.1, CI95: 66.6, 83.6). However, the heterogeneity was high (I² = 98.7%), which may be due to the large variety in the evaluations and experimental conditions.

According to these results, it can be concluded that neutrophils have a pronociceptive role in the acute pain models studied. Accordingly, treatment with fucoidan can be considered an interesting strategy for the treatment of pain in painful pathologies that involve an acute neutrophil response.

In addition, an important variable that can influence the analgesic efficacy is the administration route, as the bioavailability of fucoidan may vary between administration routes. Most of the studies employed the intravenous route 26/36, 9/36 of the studies used the intraperitoneal route, and only 1/36 used the oral route. A subgroup analysis stratified according to the administration route used concluded that the effect size for the analgesic efficacy was 69.06 (CI95: 61.15, 76.98) for the intravenous route, 87.85 (CI95: 65.86, 109.84) for the intraperitoneal route, and 115.87 (CI95: 103.75, 127.99) for the oral route. These results suggest that the intraperitoneal and oral bioavailability of fucoidan is high as the efficacy was equivalent to the one achieved by intravenous administration. Another variable which may influence the analgesic efficacy is the source of fucoidan, as its composition may vary between sources. However, the vast majority of studies employed the same commercial fucoidan extracted from *Fucus vesiculosus* (Sigma-Aldrich, St. Louis, MO, USA), so it was not necessary to conduct this analysis.

### 2.5. Fucoidan as a Tool to Study Opioid-Related Endogenous Analgesia

Endogenous analgesia has a very relevant role in the pathophysiology of pain and its treatment [100,101]. Among the different proposed mechanisms, the endogenous opioid system is one of the main players [102,103,104]. In fact, it is known to be involved in many well-known phenomena, such as the placebo effect [105]. Immune cells are the main actors in these mechanisms due to their ability to produce and release endogenous opioid peptides (EOPs) in response to different situations. As neutrophils are the primary cells during early inflammation, they constitute the main source of EOPs in the periphery.

Information on studies using fucoidan to evaluate endogenous opioid analgesia can be found in Table 2. These articles employed fucoidan together with an experimental analgesic strategy (e.g., ankle joint mobilization or light-emitting diode therapy) in order to evaluate the participation of neutrophils in the efficacy of the intervention being tested. In other words, in these studies, fucoidan was only a tool for evaluating if the analgesic efficacy of the experimental compound/intervention was dependent on OEP liberation by the recruited neutrophils. Already in 1998, the first study using fucoidan demonstrated that it was capable of blocking endogenous opioid analgesia associated with the release of EOP by immune cells (mainly neutrophils) after a stressful situation (cold water swim) [69]. A few years later, the same authors confirmed that the abolishment of endogenous opioid analgesia with the treatment of fucoidan was mediated by P-selectin and L-selectin inhibition, with the decrease in neutrophils and the consequent halving of EOP being confirmed by cytometry [61]. Subsequently, other authors have replicated these experiments with similar results, while others used this tool to elucidate whether the effect of an analgesic treatment was mediated by POEs [106,107,108]. In parallel, other authors have confirmed the effect of fucoidan in the reduction of opioid analgesia (induced by stress or corticotropin-releasing factor administration), employing other strategies for neutrophil depletion, such as anti-Ly6-G antibodies [17,22,109,110,111].

### 2.6. Fucoidan for Treating Pain-Related Conditions: Preclinical Studies

The previous analyses evaluated the effect of feeling pain after a pretreatment with fucoidan. Therefore, it is interesting to evaluate its analgesic efficacy when the treatment is therapeutic (once the pathology is already established) instead of prophylactic. Several studies evaluating the therapeutic use of fucoidan in pain-related diseases were found, and they are summarized in Table 3. It is interesting to highlight a good analgesic efficacy observed in two different models of neuropathic pain (nerve injury [112] and chemotherapy-induced injury [113]), in models of visceral inflammation (colitis [114], prostatitis [31]), and in models of joint degeneration [32].

### 2.7. Fucoidan for Treating Pain-Related Conditions: Human Studies

The promising preclinical results mentioned earlier prompted the initiation of various small to medium-sized clinical trials in humans to assess the effectiveness of fucoidan in treating pain-related conditions (summarized in Table 4). These studies were mainly performed in patients with joint pain associated with osteoarthritis, and promising results were observed. In addition to the anti-neutrophil effect, which may explain these positive results in pain and joint degradation, fucoidan has also demonstrated antifibrotic efficacy (specifically in the joint) by inhibiting the differentiation of fibroblast-like synoviocytes into myofibroblasts with tumor cell-like characteristics and restoring apoptosis [116]. Also, fucoidan was capable of ameliorating intervertebral disc degeneration in a preclinical model by restoring redox and matrix homeostasis of nucleus pulposus [32].

### 2.8. Deleterious Effects of Neutrophil Depletion

Neutropenic mice were found to have a larger quantity of large-sized *Candida auris* abscesses, suggesting an inability of the adaptive immune response to clear fungal infections in the absence of neutrophils [120], which may even cause death in the host [97]. However, it has been proposed that neutrophils are not that crucial for other infective pathologies [96]. A recent study found that early neutrophil depletion following an inflammatory injury could contribute to pain becoming chronic [121]. This suggests that an early neutrophilic response plays a protective role in preventing pain chronicity. Many authors have demonstrated that neutrophils are crucial cells in tissue regeneration processes, implying that inhibiting them could hinder tissue repair [122]. However, this notion is controversial, as other authors suggest the opposite—that neutrophils may cause further tissue damage, leading to delayed healing and excessive scar formation [123].

### 2.9. Disadvantages of Using Fucoidan for Neutrophil Depletion

The lack of specificity of fucoidan towards neutrophils in experimental setups might introduce ambiguity in delineating the pronociceptive role of neutrophils in pain, considering the potential involvement of other inflammatory cell types. Nevertheless, this circumstance could present a favorable prospect for fucoidan as a therapeutic intervention, as its anti-inflammatory and analgesic properties might be enhanced, given the reported pronociceptive nature of other inflammatory cell populations. Another disadvantage is that the composition and structure of fucoidan is dependent on seaweed species, geographic location, harvesting season, anatomical regions, and extraction procedures [41,42,45,46,47,48,49,50,51]. This structural variability (with molecular weight ranging from 10,000 to 100,000 Da) may generate variability in the bioactivity of fucoidan [42,45,124,125]. In addition, it is well known that fucoidan produces anticoagulant effects [126,127], which my interfere in its analgesic efficacy. In this line, some related adverse events, such as bleeding, may be associated with the fucoidan treatment.

## 3. Methods

### 3.1. Protocol and Registration

The methodology used in this review was specified in advance and documented in a protocol registered on Prospective Register of Systematic Reviews (PROSPERO) under the registration ID CRD42024534839. This review was performed in accordance with the latest version (2020) of the Preferred Reporting Items for Systematic Reviews and Meta-Analyses (PRISMA) guidelines on systematic reviews and meta-analyses [128].

### 3.2. PICO Research Question

(P) Animal models of pain.

(I) Pretreatment with fucoidan, also known as fucoidin.

(C) Control group treated with vehicle.

(O) Changes in pain-related parameters.

(S) Original preclinical studies.

### 3.3. Information Sources and Search Strategy

We performed a comprehensive systematic search in PubMed without restrictions for the language or date. The search was updated on 25 May 2024. The search strategy was as follows: (fucoidan OR fucoidin) AND ((pain OR hyperalgesia OR analgesia OR pain* OR nocicept* OR allodynia OR hyperalges*) OR (neutrophil* OR polymorphonuclear OR Ly6G OR GR-1)).

### 3.4. Inclusion and Exclusion Criteria

Inclusion criteria: original preclinical studies using animal models of pain in which the effect of the pretreatment (before injury) with fucoidan regarding pain outcomes was assessed.

Exclusion criteria: non-pain model, absence of pain measurement, review articles, human studies, in vitro or ex vivo experiments, studies including no relevant information or missing information, and violations of any of the above inclusion criteria.

### 3.5. Article Selection

Titles and abstracts of studies were retrieved using the search strategy by two review authors (MAH and MAT) in a blind manner to identify studies that potentially met the inclusion criteria. The full texts of these potentially eligible studies were retrieved and independently assessed for eligibility by the same team members (MAH and MAT). The selection process was carried out using the software Rayyan. Any disagreement between the authors was resolved through a discussion with a third reviewer (FRN).

### 3.6. Data Extraction

MAH and MAT independently extracted data from each included article by using a standardized, pre-piloted form. Discrepancies were identified and resolved through discussion (with a third author (FRN). Numerical data were extracted from tables, text, or figures. When they were not reported, data were extracted from graphs using digital ruler software. In case data were not reported or unclear, we contacted authors by e-mail. In case an outcome was measured at multiple time points, data from the time point where efficacy was highest were included. Extracted information included study setting; study population (animal model of pain used, including species and strains) and baseline characteristics; details of the fucoidan intervention, the timing, the dose used, and control conditions; study methodology; pain assessment test used; and main results of the intervention.

### 3.7. Meta-Analysis and Statistics

Meta-analyses were conducted using the *metafor* package in R, version 4.1.2 [129,130]. Since different pain outcomes were measured with different scales in the included studies, outcome data were normalized using the normalized mean difference (NMD), which is a useful approach when conducing a meta-analysis of preclinical data because it relates the magnitude of effect in the treatment group to a group of healthy animals [131]. Then, a 95% confidence interval (CI95%) was computed. The inverse variance statistical analysis method was used to summarize the effect sizes of the treatment, and the combined results were analyzed using the random effect model, which accounted for the variance within and between studies [132]. Effects were considered statistically significant when the p value was less than 0.05. For assessing heterogeneity, the Cochrane’s Q test (with *p* < 0.10 indicating asymmetry) and the Higgins–Thompson I^2^ values (null or low, 0–30%; medium, 30–50%; moderate, 50–75%; and high heterogeneity, >75%) were used to assess the heterogeneity within the pooled studies [133].

## 4. Concluding Remarks

Fucoidan is an interesting preclinical tool for depleting neutrophils, which may be useful to study their role in pain and other diseases. Moreover, in preclinical studies, preventive treatment with fucoidan, which substantially reduced neutrophil infiltration, was very effective for reversing acute pain. Furthermore, the therapeutic treatment with fucoidan also showed a robust analgesic effect in models of neuropathic, visceral, and joint pain. These positive preclinical results led to the launch of small clinical trials which reported some positive results, mainly for joint pain associated with osteoarthritis, with a good safety profile. Based on our data, fucoidan may be an interesting analgesic strategy for some pair-related conditions. However, randomized placebo-controlled trials involving more patients should be performed to confirm these promising findings.

## Figures and Tables

**Table 1:** Information on studies using fucoidan to evaluate the role of neutrophils in pain.

Study	Species; Strain	Dose; Route; Administration Time before Injury; Positive Control	Neutrophil Count; Efficacy/Selectivity, % Neutrophil Depletion	Pain Model	Pain Outcome	Analgesic Efficacy (*p* > 0.05)
Machelsa et al., 1998 [69]	Rat; Wistar	10 mg/kg; i.v.; 10 min; fentanyl	Manual count (β-endorphin) (≈40%)	CFA (i.pl.)	MH	No
* Dell’Antonio et al., 2002 [70]	Rat; Wistar	10 mg/kg; i.v.; 30 min; oxidized ATP	Non-evaluated	CFA (i.pl.)	MH	No
Tambeli et al., 2006 [71]	Rat; Wistar	20 mg/kg; i.v.; 30 min; tropisetron, indomethacin, and atenolol	MPO; yes/no (≈60%)	5-HT (i.pl.)	NB (Paw)	Yes
Lavich et al., 2006 [90]	Rat; Wistar	10 mg/kg, i.v.; 15 min; anti-neutrophil serum	Manual count and MPO; (>90%)	Ovalbumin probe in sensitized rats	HH	Yes
Oliveira et al., 2007 [72]	Rat; Wistar	20 mg/kg, i.v.; 20 min; tropisetron, indomethacin, guanethidine, and atenolol	Non-evaluated	5-HT; PGE; epinephrine (i.pl.)	MH	Yes; no; no
Cunha et al., 2008 [68]	Rat; Wistar	20 mg/kg; i.v.; 10 min; no positive control	MPO; yes/no (=90%)	Carrageenan (i.pl.)	MH	Yes
Guerrero et al., 2008 [77]	Mouse; C57BL/6	20 mg/kg; i.v.; 20 min; indomethacin, MK886, celecoxib, anti-neutrophil antibody	MPO; yes/yes	LTB4; zymosan (i.a.)	MH	Yes
Russell et al., 2009 [64]	Mouse; CD1	40 mg/kg; i.v.; 20 min; SB366791, TNF inhibitor, PKC inhibitor, indomethacin, COX2 inhibitor, and nimesulide	MPO; yes/no (=70%)	TNF (i.pl.)	HH	Yes
Verri et al., 2009 [65]	Mouse; BALB/c	20 mg/kg; i.v.; 15 min; bosentan, BQ123, and BQ788	MPO; yes/no (=70%)	Carrageenan (i.pl.)	MH	Yes
Marotta et al., 2009 [91]	Rat; Wistar	10 mg/kg; i.v.; 15 min; WEB2086, anti-TNF, IL-1 antagonist, indomethacin, and celecoxib	MPO; yes/no (=90%)	PAF (i.pl.)	MH; NB (Paw)	Yes; no
McNamee et al., 2010 [92]	Mouse; C57BL/6	20 mg/kg; i.p.; 24 h before every 2 d; anti-NGF, and anti-TNF	MPO; yes/no (50%)	DMM surgery; OA	NB (WB)	Yes
Pinto et al., 2010 [79]	Mouse; BALB/c	20 mg/kg; i.v.; 15 min before and 3.5 h after; infliximab, IL-1 antagonist, and DF2156	Manual count (May–Grünwald–Giemsa); yes/no (80%)	AIA-mBSA or IL-17 (i.a.)	MH	Yes
Sachs et al., 2011 [78]	Mouse; C57Bl/6J	20 mg/kg; i.v.; 10 min; lidocaine, morphine, dexamethasone, anti-TNF, and IL-1 antagonist	MPO; yes/no (>90%)	AIA-mBSA (i.a.)	MH	Yes
McNamee et al., 2011 [73]	Mouse; C57BL/6	20 mg/kg; i.p.; 2 h; anti-TNF	MPO; yes/no (>90%)	TNF; IL-17 (i.pl.)	HH; NB (WB)	Yes
Amaral et al., 2012 [80]	Mouse; C57BL/6J	20 mg/kg; i.v.; 15 min; IL-1 antagonist, DF2162, CP105,696, and MK886	MPO; yes/no (>90%)	MSU crystals (i.a.)	MH	Yes
Chatterjea et al., 2012 [62]	Mouse; ND4	20 mg/kg; p.o.; 30 min; sodium cromoglycate	MPO; Hematoxylin- Eosin; yes/no (>90%)	Compound 48/80 (i.pl.)	HH	Yes
* Oliveira-Fusaro et al., 2012 [83]	Rat; Wistar	20 mg/kg; i.v.; 20 min; tropisetron, guanethidine, atenolol, and indomethacin	MPO; yes/no (>90%)	5-HT (i.a.)	NB (Face)	No
Finley et al., 2013 [63]	Rat; Sprague Dawley	40 mg/kg; i.p.; 30 min; sphingosine-1 antagonists and fingolimod	MPO; Hematoxylin- Eosin; yes/no (>60%)	Carrageenan; S1P; SEW2871 (i.pl.)	HH	Yes
Guerrero et al., 2013 [82]	Mouse; BALB/C	20 mg/kg; i.v.; 15 min; indomethacin, MK886, selective PAFR antagonists	MPO; no/no	PAF (i.a.)	MH	Yes
Perin-Martins et al., 2013 [74]	Rat; Wistar	20 mg/kg; i.v.; 30 min; TRPA1 antagonist, CGRP antagonist, indomethacin	MPO; yes/no (100%)	Allyl isothiocyanate (i.pl.)	MH	Yes
Zarpelon et al., 2013 [75]	Mouse; BALB/C	20 mg/kg; i.v.; 15 min; infliximab, IL1 antagonist, anti-CXCL1, indomethacin, BQ788, and clazosentan	MPO; yes/no (>90%)	IL–33 (i.pl.)	MH	Yes
* Albuquerque et al., 2013 [93]	Mouse; BALB/C	20 mg/kg; i.v.; 30 min; morphine and dipyrone	Manual counting; yes/no (>90%)	Acetic acid (i.p.)	NB (writhing)	Yes
Teixeira et al., 2014 [76]	Rat; Wistar	25 mg/kg, i.v., 20 min; P2X7 and P2X1,3,2/3 antagonists, atenolol, and indomethacin	Non-evaluated	BzATP (i.pl.)	MH	Yes
Schiavuzzo et al., 2015 [87]	Rat; Wistar	25 mg/kg; i.v.; 20 min; P2X1,3,2/3 antagonist, lidocaine, bradykinin antagonist, and indomethacin	MPO; no/no	α,β-meATP (i.m.)	MH	Yes
Silva et al., 2017 [88]	Mouse; C57BL/6	20 mg/kg; i.v.; daily from 2 to 7 d; dexamethasone, morphine, lidocaine, infliximab, etanercept, clodronate, and indomethacin	Flow cytometry; yes/yes (>90% reduced CD45+ cells)	HSV-1 (postherpetic neuralgia)	MA	Yes
Santos et al., 2017 [66]	Rat; Wistar	25 mg/kg; i.v.; 20 min; dexamethasone, bradykinin antagonist, atenolol, indomethacin, and ICI118	MPO; no/no	Sustained muscle contraction	MH	Yes
Dornelas-Filho et al., 2018 [94]	Mouse; Swiss	100 mg/kg; i.v.; 30 min; anti-neutrophil antibody	MPO; flow cytometry (>90%)	Ifosfamide-induced hemorrhagic cystitis	MH	Yes
Zanelatto et al., 2018 [84]	Rat; Wistar	20 mg/kg; i.v.; 20 min; propranolol and thalidomide	MPO; no/no	Isoproterenol (i.a.)	NB (Face)	Yes
Yin et al., 2020 [81]	Mouse; C57BL/6J	20 mg/kg; i.v.; 8 h y 23 h after; clodronate	MPO; yes/no (>95%)	MSU crystals (i.a.)	MA	Yes
Teixeira et al., 2020 [86]	Rat; Wistar	25 mg/kg, i.v.; 30 min; P2X3 and P2X2/3 antagonist	MPO; yes/no (100%)	αβ-meATP (i.a.)	NB (WB)	Yes
Teixeira et al., 2021 [85]	Rat; Wistar	25 mg/kg; i.v.; 20 min; P2X7 antagonist, lidocaine, atenolol, and indomethacin	Manual count; no/no	BzATP (i.a.)	NB (Face)	Yes

Abbreviations: 5-HT: 5-hydroxytryptamine; α,β-meATP: α,β-methylene ATP; AIA: antigen-induced arthritis; BzATP: 3′-O-(4-Benzoyl)benzoyladenosine 5′-triphosphate; CFA: complete Freund’s adjuvant; DMM: destabilization of the medial meniscus; Face: facial grooming; HH: heat hyperalgesia; HSV-1: herpes simplex virus type 1; i.a.: intra-articular; IL-17: interleukin 17; i.m.: intramuscular; i.p.: intraperitoneal; i.pl.: intraplantar; i.v.: intravenous; LTB4: leukotriene B4; MA: mechanical allodynia; mBSA: methylated bovine serum albumin; MH: mechanical hyperalgesia; MPO: myeloperoxidase assay; MSU: monosodium urate; NB: nociceptive behavior; PAF: platelet-activating factor; p.o.: oral; Paw: paw flinching; S1P: sphingosine-1-phosphate; TNF: tumor necrosis factor; WB: weight bearing distribution. * Articles not included in this meta-analysis as one of the comparison groups was missing.

**Table 2:** Information on studies using fucoidan to evaluate endogenous opioid analgesia.

Study	Species; Strain	Fucoidan Dose; Route; % Neutrophil Reduction	Pain Model	Pain Outcome	Main Results
Machelsa et al., 1998 [69]	Rat; Wistar	10 mg/kg; i.v.; ≈40% indirect reduction (β-endorphin)	CFA (i.pl.)	MH	Decreased analgesia induced by cold water swim stress and CRF
Machelsa et al., 2004 [17]	Rat; Wistar	10 mg/kg; i.v.; ≈50% (macrophage and T cell reduction)	CFA (i.pl.)	MH	Abolished peripheral stress-induced antinociception
Martins et al., 2012 [106]	Mouse; Swiss	100 µg/mouse; i.p.; non-evaluated	PI	MH	Did not reverse analgesia induced by ankle joint mobilization
Cidral-Filho et al., 2013 [107]	Mouse; Swiss	100 μg/mouse; i.p.; non-evaluated	PI	MH	Abolished the analgesic effect of light-emitting diode therapy
Martins et al., 2016 [108]	Mouse; Swiss	100 μg/mouse; i.p.; non-evaluated	CFA (i.pl.)	MH	Abolished the analgesic effect of light-emitting diode therapy

Abbreviations: CFA: complete Freund’s adjuvant; CRF: corticotropin-releasing factor; i.p.: intraperitoneal; i.pl.: intraplantar; i.v.: intravenous; MH: mechanical hyperalgesia.

**Table 3:** Therapeutic use of fucoidan in pain-related diseases.

Study	Pathological Model	Intervention; Positive Control	Main Results
Zhang et al., 2001 [114]	Dextran sodium sulfate-induced murine colitis	Fucoidan daily (25 mg/kg, i.v.) starting immediately prior to the 5-day challenge; DF2162 and MK886 and CP105,696	Fucoidan can reduce mucosal damage and crypt destruction in the colon of dextran sodium sulfate-treated mice, relieving chronic colitis.
Hu et al., 2014 [112]	Peripheral nerve injury produced by spinal nerve ligation	Fucoidan (15, 50 and 100 mg/kg, i.t.) once daily during the period of days 11–20, inclusively; no positive control	Attenuated the existing allodynia and hyperalgesia induced by nerve injury. Also inhibited cytokines production, glial. and ERK activation.
Hu et al., 2017 [113]	Chemotherapy-induced peripheral neuropathy (vincristine)	Fucoidan (50, 100 or 200 mg/kg, i.p.) on single treatment (day 14) or repeated treatment once daily for 14 days; pregabalin	Repeated. However, no single treatment attenuated vincristine-induced mechanical and cold allodynia in a dose-dependent manner.
Wang et al., 2018 [33]	Peripheral arterial disease by injection of sodium laurate into femoral artery	Low molecular weight fucoidan (20, 40 or 80 mg/kg/day; p.o.) for 4 weeks; cilostazol	Ameliorated foot ulceration and improved plantar perfusion. Suppressed the upregulation of inflammatory factors (ICAM-1 and IL-1β) in the gastrocnemius muscles of ischemic hindlimb.
Ahn et al., 2019 [34]	Transient global cerebral ischemia in obese gerbils	Fucoidan (50 mg/kg; p.o.) daily for the last 5 days; no positive control	Relieved acceleration and exacerbation of ischemic brain injury in an obese state via the attenuation of obesity-induced severe oxidative damage.
Obluchinskaya et al., 2021 [115]	Peripheral inflammation carrageenan (i.pl.)	Fucoidan-based cream 100, 200, and 400 mg/rat/day (topical) for 5 days; diclofenac	Inhibited carrageenan-induced edema and ameliorated mechanical allodynia (efficacy comparable with diclofenac).
Zhang et al., 2022 [31]	Experimental autoimmune prostatitis	Fucoidan 20 mg/kg (i.p.) one day before disease induction and then once a week; anti-neutrophil antibody	Histological appearance of prostate tissues improved, and chronic pain development was ameliorated.
Li et al., 2023 [32]	Intervertebral disc degeneration	Single treatment with 5 μL of fucoidan (10 μg/mL; intra-disc injection); no positive control	Ameliorated intervertebral disc degeneration 4 and 8 days after injury, and preserved disc height, extracellular matrix components, and nucleus pulposus hydration.

Abbreviations: ERK: Extracellular-Signal-Regulated Kinase; ICAM-1: Intercellular Adhesion Molecule 1; IL-1β: interleukin 1β; i.p.: intraperitoneal; i.pl.: intraplantar; i.v.: intravenous; p.o.: oral administration.

**Table 4:** Human studies evaluating fucoidan for pain-related diseases.

Study	Study Type; Population	Intervention; Route; Dosage	Main Efficacy Results	Main Safety Results
Myers et al., 2010 [117]	Open-label, randomized, combined Phase I and II; knee osteoarthritis (*n* = 10 adults 9F/1M)	100 and 1000 mg of fucoidan; p.o.; daily for 4 weeks	Reduced COAT score by 18% for the 100 mg treatment and 52% for the 1000 mg dose. Clear dose response in all COAT subscales (pain, stiffness, physical activity and overall symptom severity)	Well tolerated (few adverse events related to treatment). No changes in blood parameters
Myers et al., 2016 [40]	Randomized placebo-controlled trial; hip/knee osteoarthritis (*n* = 96 adults 56F/40M)	300 mg dose of a *Fucus vesiculosus* extract (85% fucoidan); p.o.; daily for 12 weeks	Fucoidan improved by 29% COAT score (knee), while placebo improved it by 30.6%. No significant differences in symptom reduction vs. placebo. No difference in the usage of paracetamol	It was safe and well tolerated
Kan et al., 2020 [118]	Randomized, double-blind, placebo-controlled trial; chronic gastritis (*n* = 101 adults)	Wheat peptides and fucoidan; p.o.; once daily for 45 days	Reduced gastric mucosal damage in 70% of subjects. Significantly less stomach pain, belching, bloating, acid reflux, appetite loss, increased food intake, and higher quality of life (*p* > 0.05 for all)	No adverse event reported
Tay et al., 2022 [119]	Randomized, double-blind, placebo-controlled trial; prediabetes and hip or knee joint pain (*n* = 150 adults)	20 g of chocolate (1000 mg mussel powder and 1000 mg of fucoidan); p.o.; once daily for 100 days	Results not available; the primary endpoints are change in insulin resistance and patient-reported joint pain. Secondary endpoints include anthropometry, fasting glucose and insulin, HbA1c, inflammatory markers, satiety, quality of life, physical function, pain intensity, and analgesic medication use	Results not available; complications reported and described by duration, severity, outcome, treatment, and relation to study treatment or cause

Abbreviations: COAT: comprehensive arthritis test; p.o.: oral administration.

## Data Availability

The raw data supporting the conclusions of this article will be made available by the authors upon request.
